# Immune Activations and Viral Tissue Compartmentalization During Progressive HIV-1 Infection of Humanized Mice

**DOI:** 10.3389/fimmu.2019.00340

**Published:** 2019-02-28

**Authors:** Hang Su, Yan Cheng, Sruthi Sravanam, Saumi Mathews, Santhi Gorantla, Larisa Y. Poluektova, Prasanta K. Dash, Howard E. Gendelman

**Affiliations:** ^1^Department of Pharmacology and Experimental Neuroscience, College of Medicine, University of Nebraska Medical Center, Omaha, NE, United States; ^2^Department of Pharmaceutical Sciences, College of Pharmacy, University of Nebraska Medical Center, Omaha, NE, United States

**Keywords:** humanized mice, host immune responses, HIV-1 seeding, viral tissue compartments, immune activation, host inflammatory responses

## Abstract

Human immunodeficiency virus type one (HIV-1) tissue compartments are established soon after viral infection. However, the timing in which virus gains a permanent foothold in tissue and the cellular factors that control early viral-immune events are incompletely understood. These are critical events in studies of HIV-1 pathogenesis and in the development of viral reservoirs after antiretroviral therapy. Moreover, factors affecting the permanence of viral-tissue interactions underlie barriers designed to eliminate HIV-1 infection. To this end we investigated the temporal and spatial viral and host factors during HIV-1 seeding of tissue compartments. Two humanized NOD.Cg-Prkdc^scid^ IL2rg^tm1Wjl^/SzJ mouse models were employed. In the first, immune deficient mice were reconstituted with human CD34+ cord blood hematopoietic stem cells (HSC) (hu-HSC) and in the second mice were transplanted with adult mature human peripheral lymphocytes (hu-PBL). Both, in measure, reflect relationships between immune activation and viral infection as seen in an infected human host. Following humanization both mice models were infected with HIV-1_ADA_ at 10^4^ 50% tissue culture infective doses. Viral nucleic acids and protein and immune cell profiles were assayed in brain, lung, spleen, liver, kidney, lymph nodes, bone marrow, and gut from 3 to 42 days. Peripheral CD4+ T cell loss began at 3 days together with detection of HIV-1 RNA in both mouse models after initiation of HIV-1 infection. HIV-1 was observed in all tested tissues at days 3 and 14 in hu- PBL and HSC mice, respectively. Immune impairment was most prominent in hu-PBL mice. T cell maturation and inflammation factors were linked directly to viral tissue seeding in both mouse models. We conclude that early viral tissue compartmentalization provides a roadmap for investigations into HIV-1 elimination.

## Introduction

Following the introduction of antiretroviral therapy (ART) in the mid-1990s, remarkable progress was made toward reducing disease morbidities and mortality during a life-long human immunodeficiency virus type one (HIV-1) infection (1–3). While ART efficiently controls viremia and preserves immune function (4) it does not eradicate infection (5). Recent discoveries suggested that HIV-1 persistence is established within 2 weeks of viral exposure (6). Thus, complete understanding of viral tissue compartmentalization needs be made in efforts to eliminate HIV-1 infection.

To reflect the temporal and spatial challenges of human infection, animal models must reflect essential features of HIV-1 pathobiology in its human host (7). Insights into HIV-1 transmission and tissue distribution were made through studies of simian immunodeficiency virus (SIV) infection of nonhuman primates (8, 9). However, there are limitations. *First*, SIV and HIV are genetically and biologically distinct (10). *Second*, divergent viral and host factors affect progression to the acquired immune deficiency syndrome which commonly occurs more rapidly during SIV than HIV (10). Therefore, an HIV-1 susceptible animal model would be preferable for studies that reflect human infection. To such ends, humanized mouse models were developed. These models received engraftment of human cells into immunodeficient rodents resulting in the establishment of a functional human immune systems and tissue microenvironment that support long-term HIV-1 replication in target cells and tissues (11). Studies conducted by our group and others using such humanized mice have provided new insights into HIV-1 virology, immunology, pathology, therapeutics, and modes of viral eradication (12–16). However, to date, limited studies were performed to dissect when and to what extent HIV-1 establishes persistent infection in tissue compartments. If this information is gleamed they could prove instrumental in developing improved antiretroviral therapies.

In our prior works, chronic HIV-1 infected CD34+ hematopoietic stem cell (hu-HSC) reconstituted NOD.Cg-Prkdc^scid^ Il2rg^tm1Wjl^/SzJ (NSG) mice were investigated (17–19). They were used successfully to identify viral replication patterns and virus-induced injuries in diverse cell and tissue types. In the current study, attempts were made to better understand the temporal and spatial dynamics of viral seeding that followed HIV-1 inoculation. To this end we tracked early viral footprints in tissue compartments. To compare how the host microenvironment could affect viral seeding we used both infected adult peripheral blood lymphocyte (hu-PBL) and hu-HSC mouse models. Animals were evaluated in parallel after infection and were necropsied at days 3, 5, 7, 14, 28, and 42. Results showed that peripheral CD4+ T cells decreased rapidly in infected hu-PBL mice with viral detection in all tissues within 3 days of infection. In contrast, in hu-HSC mice virus was detected in gut, kidney, spleen, lung, liver, and lymph nodes and in brain only by 14 and 28 days. HIV-1 nucleic acids and proteins demonstrated that the viral life cycle was completed in both humanized mice. Transcriptomic analysis demonstrated substantive immune activation and pro-inflammatory signature in hu-PBL compared to HSC mice that paralleled viral tissue compartmentalization. These data, taken together, demonstrate the dynamics and extent of HIV-1 tissue infections and its link to human immunity in relevant humanized mouse models of viral infection.

## Materials and Methods

### Generation and HIV-1 Infection of Humanized Mice

NSG mice were purchased from the Jackson Laboratory (Bar Harbor, ME) and housed under pathogen-free conditions in accordance with ethical guidelines for the care of laboratory animals at the National Institutes of Health and the University of Nebraska Medical Center. All experimental protocols were approved by the University of Nebraska Medical Center Institutional Animal Care and Use Committee (IACUC).

To generate human CD34+ mice, the new born NSG mice were irradiated with a RS 2,000 biological irradiator (Rad Source Technologies Inc.), followed with intrahepatic engraftment of human CD34+ HSCs that were isolated from human cord blood. Humanization of the animals was monitored monthly from peripheral blood using flow cytometry analysis on human cell markers. At 20–22 weeks of age, a total of 31 animals with replicate levels of human cell reconstitution were selected then divided into uninfected (*n* = 5) and HIV-1 infected mouse groups (*n* = 26). The latter animals were infected intraperitoneally with HIV-1_ADA_ at 10^4^ TCID_50_ and then randomly distributed into groups that were sacrificed at days 3 (*n* = 5), 5 (*n* = 5), 7 (*n* = 5), 14 (*n* = 5), 28 (*n* = 3), and 42 (*n* = 3) post viral challenge for further immune and viral analysis.

Adult human PBL mice were generated by intraperitoneal injection of adult human peripheral blood lymphocytes purified from HIV-1 seronegative donor leukopaks into 8-week old NSG mice at 25 × 10^6^ PBLs/mouse. Ten days after engraftment, animal humanization was confirmed by flow cytometry. In total, 28 mice with replicate numbers of engrafted human cells were divided into uninfected (*n* = 4) and HIV-1 infected groups (*n* = 24) used for analyses. HIV-1_ADA_ challenge was given intraperitoneally at 10^4^ TCID_50_. Infected animals were then randomly distributed into groups that were sacrificed at days 3, 5, 7, and 17 (*n* = 6, 5, 5, and 8) after viral infection for further immune and viral evaluations.

### Flow Cytometry

Peripheral blood was collected at designated time points into EDTA-coated tubes by cardiocentesis at the study end. Cellular phenotypes were analyzed for human antigens CD45, CD3, CD19, CD4, CD8, and CD14 (BD Pharmingen, San Diego, CA) using the fluorescence-activated cell sorting (FACS) system BD LSR2 (BD Immunocytometry Systems, Mountain View, CA) system. CD45+ human cells were gated from total lymphocytes. The percentages of CD4^+^ and CD8^+^ cells were obtained from the gate set for human CD3+ T cells. Results were analyzed using FlowJo software (BD Pharmingen, San Diego, CA).

### Viral Load Analyses

Plasma samples were isolated from animal peripheral blood by centrifugation. Plasma HIV-1 RNA levels were measured using an automated COBAS Amplicor V2.0/Taqman-48 system (Roche Molecular Diagnostics, Basel, Switzerland) as per the manufacturer's instructions.

### Nucleic Acid Extraction and Quantification

Animal tissues were homogenized using a Qiagen Tissue Lyser II followed total nucleic acids (DNA and RNA) extraction with Qiagen All Prep DNA/RNA Mini Kit (QIAGEN). Serial dilutions of HIV-1 DNA from the ACH-2 cell line, which contains one integrated viral copy per cell, served as the standard control (20). Tissue HIV-1 RNA was first reverse-transcribed to complementary DNA using a cDNA synthesis kit (Invitrogen, MA) (21). HIV-1 DNA and RNA were quantified by semi-nested real-time PCR as previously described (19). The first round of the PCR was performed on a conventional PCR machine (T100 Thermal Cycler, BioRad, CA). The products were subsequently applied to the second round real-time PCR using TaqMan fluorescent probes on an ABI Prism 7000 real-time PCR machine (Applied Biosystems, MA). The expression levels of tissue HIV-1 DNA and RNA were normalized to those for the human CD45 gene (Life Technology, CA). The sensitivity of our assay is around 10 copies.

### RNAscope

RNA scope was performed on 5-μm thick paraffin-embedded spleen sections (Advanced Cell Diagnostics, Hayward, CA) according to the manufacturer's instructions. Anti-sense HIV-1 Clade B probe designed for targeting 854–8291 base pairs of HIV-1 sequence was used for viral detection. Positive signals were expressed as single or clusters of brown dots. Human peptidylprolyl isomerase B (PPIB) was applied as controls for human genome. All the images were captured for 40X magnification.

### Immunohistochemistry

Tissue samples were collected at the time of animal autopsy, fixed with 4% paraformaldehyde, and embedded in paraffin. Tissue sections of 5-μm thickness were cut and immuno-stained with HLA-DR (clone CR3/43, 1:100, DAKO, Carpinteria, CA) and HIV-1 p24 (1:10, DAKO) antibodies. The DAKO EnVision polymer-based system was used for staining development, and all the sections were counterstained with Mayer's hematoxylin (12). Images were obtained with a Nuance EX camera fixed to a Nikon Eclipse E800 microscope using Nuance software (Cambridge Research & Instrumentation, Woburn, MA). Human HLA-DR images were taken at 20 × magnifications and HIV-1p24 images were captured at 40 × objective magnifications.

### Human mRNA Analysis of Immune Responses

Humanized mouse spleen was harvested at animal necropsy followed with total RNA isolation using an RNease Mini Kit (QIAGEN). Complementary DNA (cDNA) was generated using a cDNA synthesis kit (Invitrogen, MA) and subscribed to RT^2^ PCR arrays for T & B cell activation analysis (QIAGEN). Quantitative RT-PCR was performed on an Master cycler® ep realplex as per the manufacturer's instructions (Eppendorf) and analyzed using RT^2^ Profiler PCR Array web-based data analysis software, version 3.5 (QIAGEN). Gene networks analysis was performed using Ingenuity Pathway Analysis (QIAGEN).

### Statistical Analyses

Data were analyzed using GraphPad Prism 7.0 software (La Jolla, CA). The Student's *t*-test was used for two-group comparison. A value of *p* < 0.05 was considered statistically significantly different. All results were presented as the means ± the standard error of the mean (SEM). Fisher's Exact Test was used to validate the IPA data of spleen of each canonical pathway.

## Results

### Immune Profiles in HIV-1 Infected Humanized Mice

**Figure 1:**
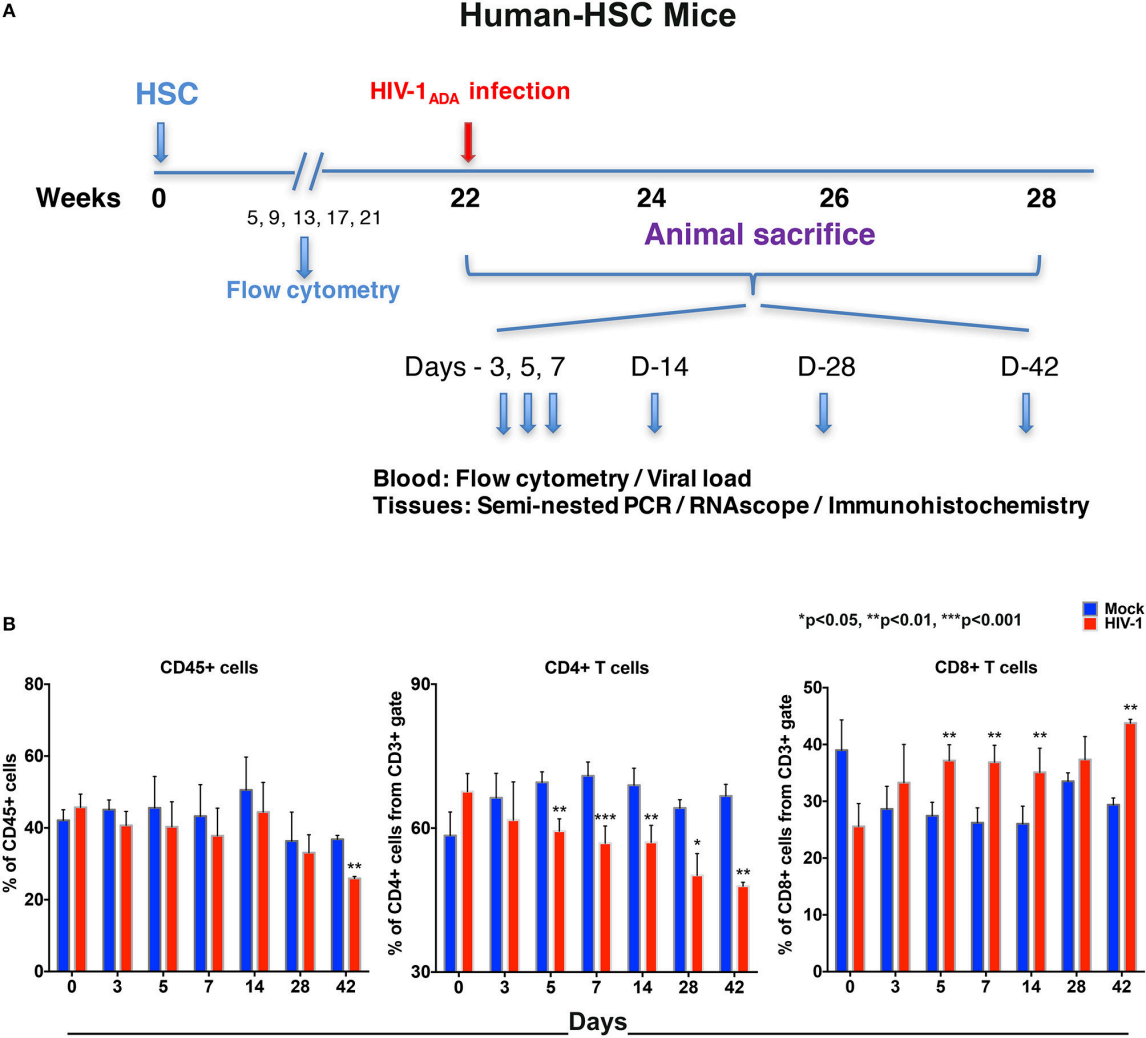
Human lymphocyte responses following HIV-1 infection of hu-HSC mice. **(A)** The illustrated experimental design for hu-HSC mice human cell reconstitution, HIV-1 infection, and serial animal sacrifice performed at days 0, 3, 5, 7, 14, 28, and 42. Animal numbers are *N* = 5, 5, 5, 5, 5, 3, and 3 at each of the time points. **(B)** Peripheral blood CD45+, CD4+, and CD8+ cell counts before/mock infection (blue) and after HIV-1 infection (red) for each of the time points by flow cytometry tests. Data are expressed as mean ± SEM and considered *, **, *** statistically different, at *p* < 0.05, *p* < 0.01, and *p* < 0.001.

NSG mice were irradiated at birth then were transplanted by intrahepatic injection with human CD34+ cord blood hematopoietic stem cells (hu-HSC) (12). Monthly whole blood flow cytometry showed that by 22 weeks mouse blood contained 30–60% human immunocytes. Following HIV-1_ADA_ infection at 10^4^ 50% tissue culture infection dose (TCID_50_)/animal, assays for viral, and immune profiles were performed in blood and tissues at days 0, 3, 5, 7, 14, 28, and 42. Replicates of 3 to 5 animals were tested at each of the time points before and after infection (at the time of sacrifice) (Figure 1A).

Our flow cytometric gating strategy is illustrated in Figure S1A. Prior to HIV-1 infection, the percentages of human CD45+ cells in hu-HSC mouse blood ranged between 30 and 60% (Figure 1B). A significant decline was seen by 42 days (10.9% ± 0.9), but not much decline was observed in the earlier time points (Figure 1B). Percentages of human cells stayed consistent between HIV-1 infected and mock infected controls in hu-HSC spleen and bone marrow that ranged from 45 to 55% (Figures S1B,C).

Progressive loss of CD4+ T cells in blood was observed in infected hu-HSC mice. The mean decreases in CD4+ T cells were 4.7% ± 5.1, 10.2% ± 2.2, 14.0% ± 0.8, 11.9% ± 1.6, 14.0% ± 2.9, and 18.8% ± 1.8, at days 3, 5, 7, 14, 28, and 42, respectively. In parallel, CD8+ T cell counts were increased by 4.6% ± 3.8, 9.7% ± 2.0, 10.6% ± 1.6, 9.1% ± 1.3, 3.8% ± 2.6, and 14.4% ± 1.1, at respective time points (Figure 1B). Splenocytes and bone marrow cells were collected at necropsy and showed parallel losses and increases in CD4+ and CD8+ T cells, respectively, in HIV-1 infected vs. mock infected mice (Figures S1B,C).

**Figure 2:**
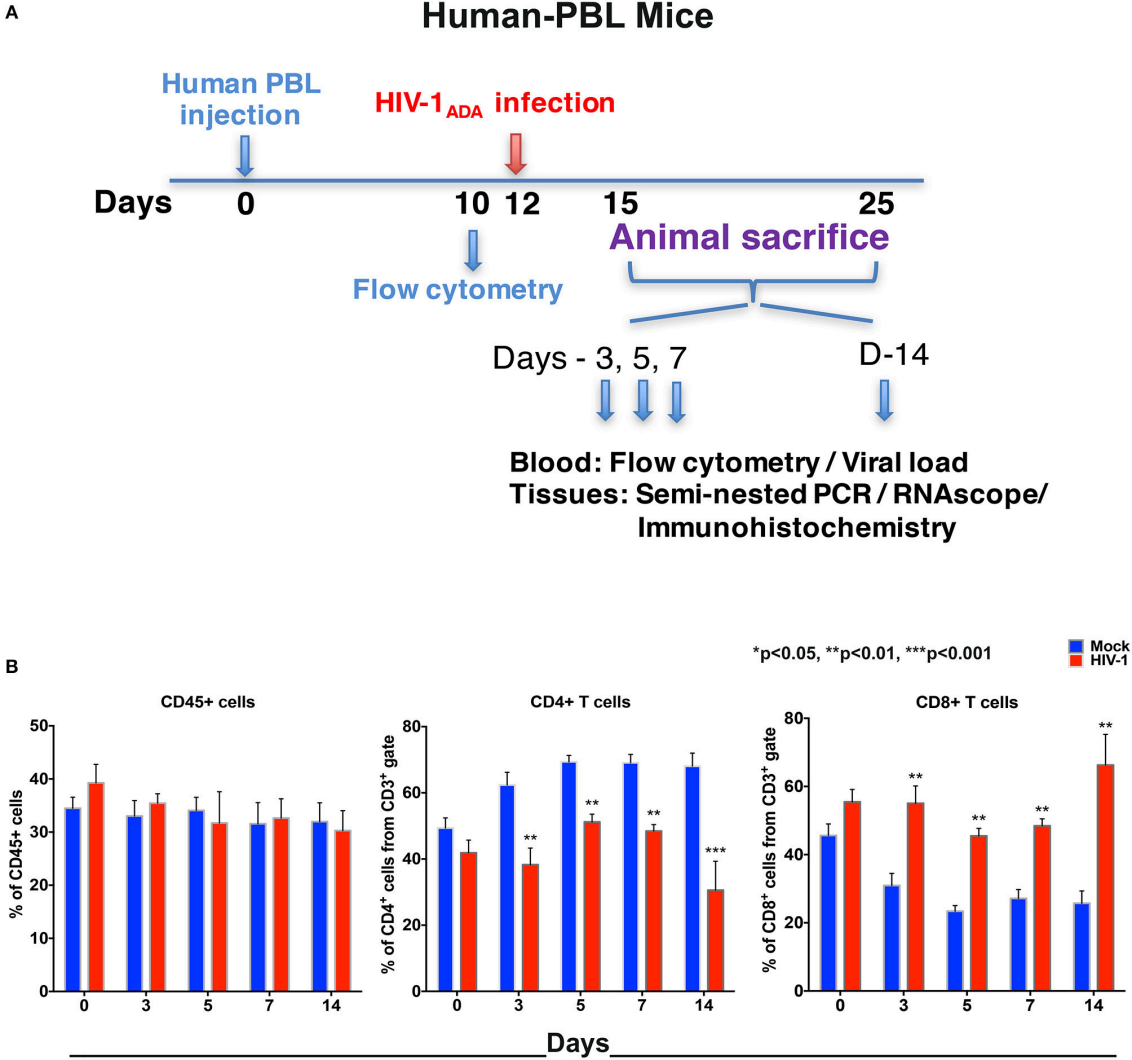
Human lymphocyte responses following HIV-1 infection of hu-PBL mice. **(A)** The illustrated experimental design for hu-PBL mice human cell reconstitution, HIV-1 infection, and serial animal sacrifice performed at days 0, 3, 5, 7, and 14. Animal number are *N* = 4, 6, 5, 5, and 8 at each of the time points, respectively. **(B)** Peripheral blood CD45+, CD4+, and CD8+ cell counts before/mock infection (blue) and after HIV-1 infection (red) for each of the time points by flow cytometry tests. Data are expressed as mean ± SEM and considered **, *** statistically significant, at *p* < 0.01 and *p* < 0.001.

To compare virus-host interactions during early HIV-1 infection with immunologically “mature” hu-PBL mice, replicate evaluations were performed. Due to expected graft-vs.-host disease in this model (22) testing was conducted up to 14 days. Adult NSG mice were engrafted with hu-PBL 10 days prior to HIV-1_ADA_ infection with up to eight animals/time point evaluated at days 0, 3, 5, 7, and 14 (Figure 2A). No significant changes of peripheral human CD45+ cell counts were observed in hu-PBL mice before and after infection. The values ranged from 25 to 45% of total immunocytes (Figure 2B). The depletion of CD4+ T cells was robust in infected hu-PBL mice. These equaled 24.1% ± 4.6, 18.2% ± 3.0, 20.6% ± 2.6, and 37.4% ± 6.9, at days 3, 5, 7, and 14, respectively, following infection. In parallel, peripheral CD8+ T cell counts rose by 24.1% ± 4.3, 22.1% ± 3.8, 21.3% ± 3.9, and 40.5% ± 7.6, at equivalent time points (Figure 2B). Taken together, the early and progressive impairment of human immune cells was observed during HIV-1 infection in both hu-HSC and hu-PBL mouse models, but more vigorously in hu-PBL than in hu-HSC mice.

### Plasma Viral Loads in HIV-1 Infected Humanized Mice

**Figure 3:**
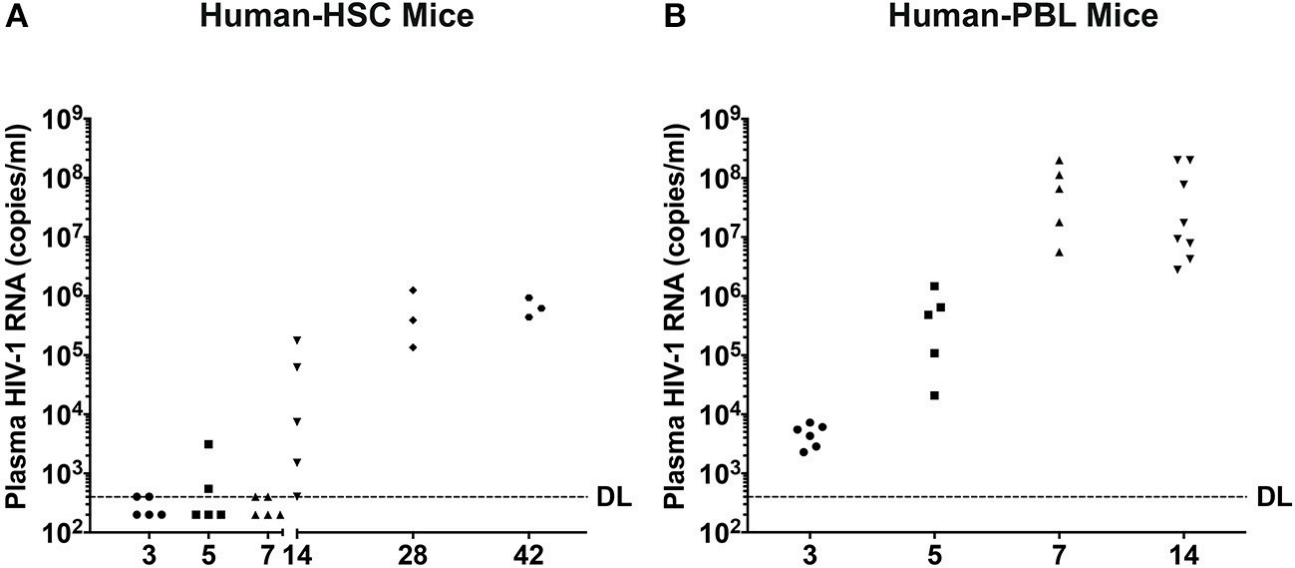
Peripheral viral loads during the course of HIV-1 infection. Plasma samples were collected following animal sacrifice from HIV-1 infected **(A)** hu-HSC and **(B)** hu-PBL mice. Fifty microliter mouse sera was collected then diluted to 1 ml with sterile filtered healthy human sera enabling a detection limit (DL) of 400 HIV-1 RNA copies/ml that is illustrated by the dashed line. Each dot represents an individual animal. The mean HIV-1 copy value from each group of animals was labeled.

HIV-1 RNA appears before antiviral antibodies in blood at 10 to 14 days after viral exposure. To recapitulate these findings blood was collected from humanized mice and analyzed for viral loads by the COBAS Ampliprep V2.0 and Taqman-48 assay (Figure 3). In hu-HSC mice, plasma HIV-1 RNA was detected in all animals with a mean of 5.0 ± 3.4 × 10^4^ copies/ml at 14 days after infection. At days 3, 5, and 7 after infection plasma viral loads were observed in 2/5 animals at or near to the detection limit of 400 copies/ml. Peak viremia was recorded at day 28 at a mean of 5.9 ± 3.4 × 10^5^ copies/ml. At 42 days plasma viral load was at 6.6 ± 1.4 × 10^5^ copies/ml (Figure 3A).

In contrast, HIV-1 RNA was readily observed in all infected hu-PBL mice at day 3 with the mean of 4.7 ± 0.8 × 10^3^ copies/ml. A 2-log increase in viral copies were observed at days 5 and 7 with means of 5.4 ± 2.6 × 10^5^ and 8.0 ± 3.5 × 10^7^ copies/ml, respectively. At day 14, plasma viral load was 8.3 ± 4.8 × 10^7^ copies/ml (Figure 3B).

### Tissue Compartments in HIV-1 Infected Humanized Mice

**Figure 4:**
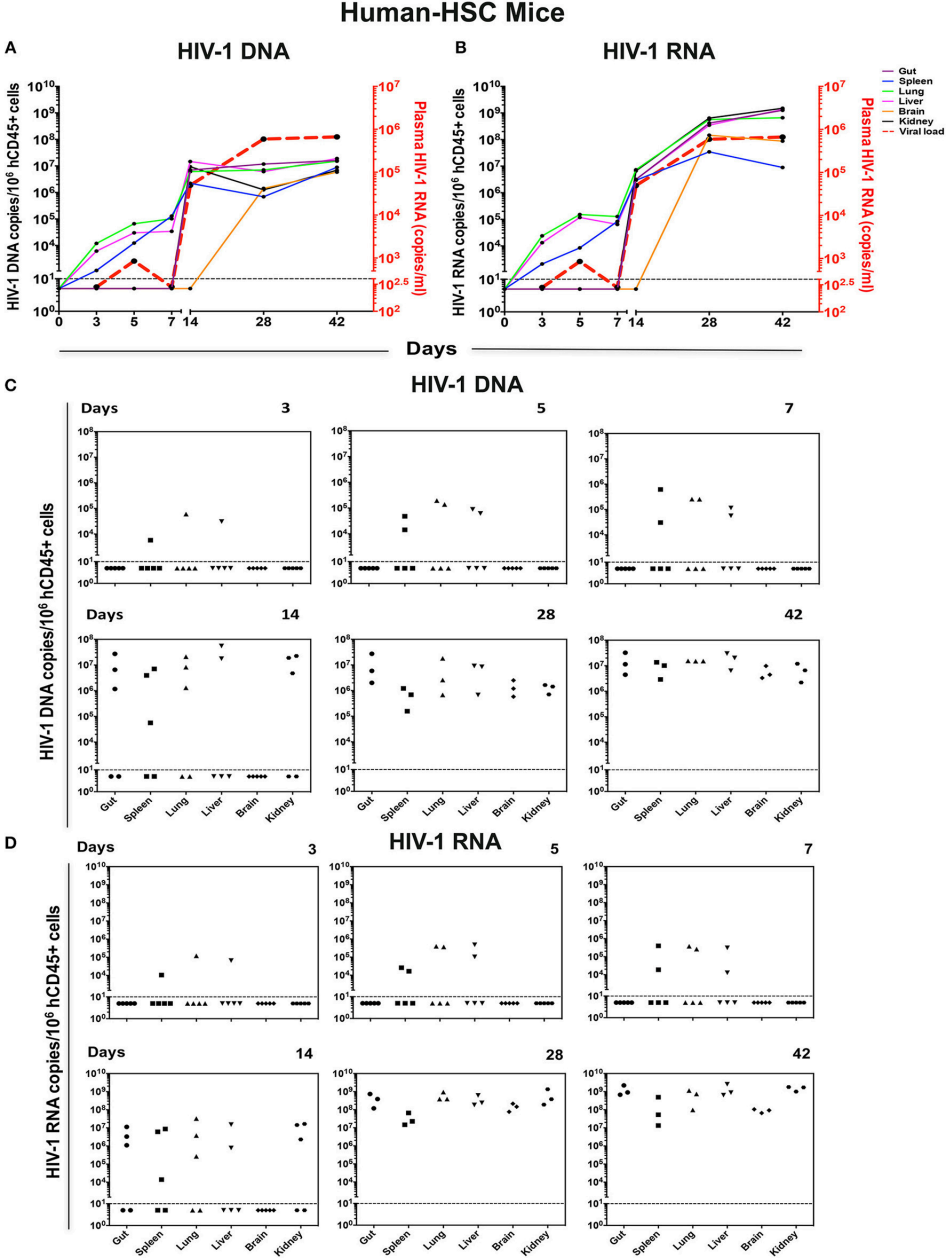
Viral tissue compartments in HIV-1 infected hu-HSC mice. Gut, spleen, lung, liver, brain, and kidney tissues were collected at necropsy at times indicated from HIV-1 infected hu-HSC mice, followed by assay of viral DNA and RNA by qPCR. The kinetics of **(A)** HIV-1 DNA and **(B)** HIV-1 RNA in each tissue are shown by colored straight lines assigning viral copies/10^6^ hCD45+ cells (left Y axis) vs. time (X axis). The temporal relationship of viral load (acquired from Figure 3A) was plotted in red dashed line (right Y axis). Data are expressed as the means. **(C)** HIV-1 DNA and **(D)** HIV-1 RNA in tissues at single time point were listed with each dot representing an individual animal. Values below the horizontal line indicated that viral DNA and RNA were below the DL.

**Figure 5:**
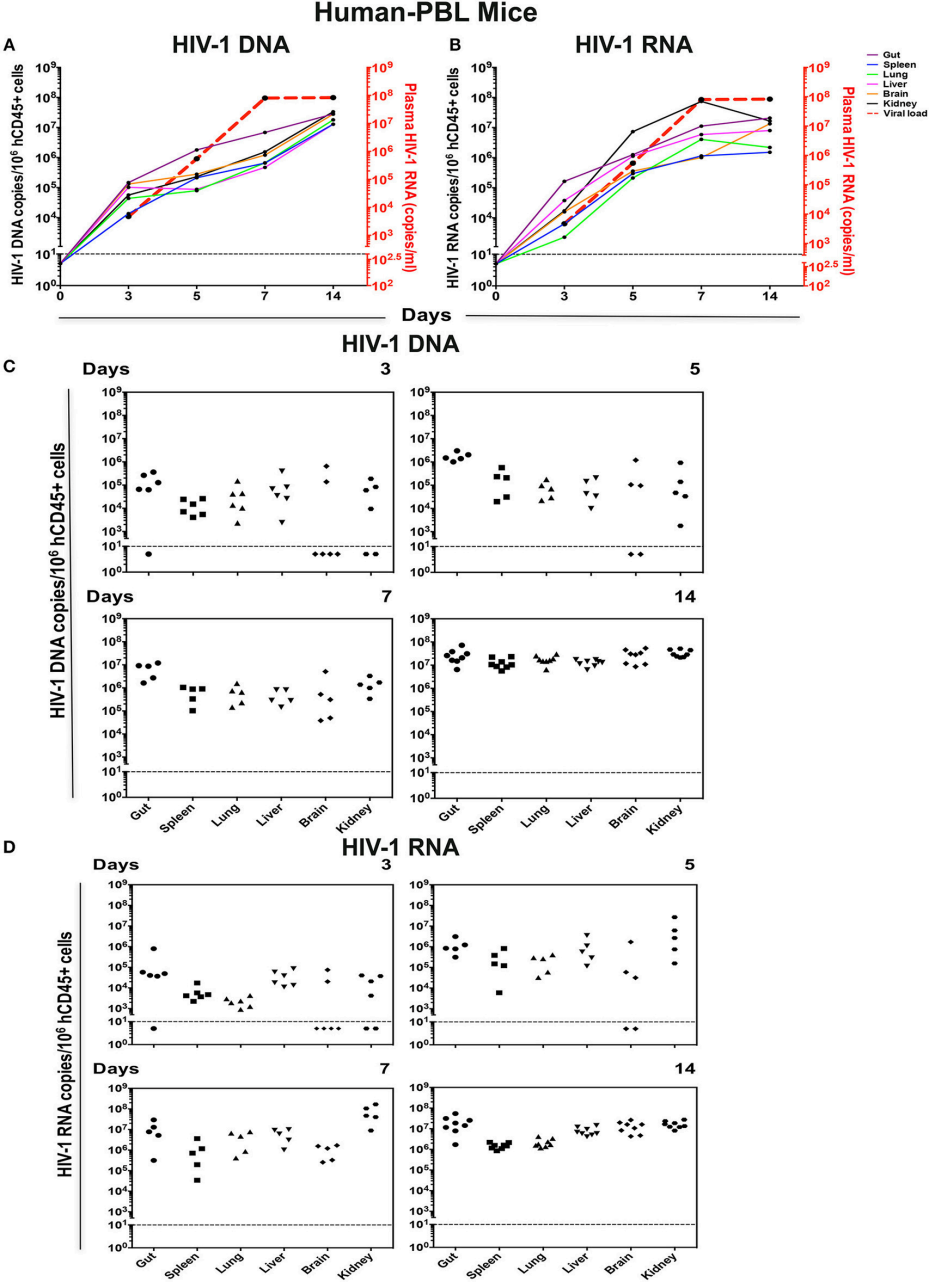
Viral tissue compartments in HIV-1 infected hu-PBL mice. Gut, spleen, lung, liver, brain, and kidney tissues were collected at necropsy at times indicated from HIV-1 infected hu-PBL mice, followed by assay of viral DNA and RNA by real-time qPCR. The kinetics of **(A)** HIV-1 DNA and **(B)** HIV-1 RNA in each tissue are shown by colored straight lines assigning viral copies/10^6^ hCD45+ cells (left Y-axis) vs. time (X axis). The temporal relationship of viral load (acquired from Figure 3B) was plotted in red dashed line (right Y axis). Data are expressed as the means. **(C)** HIV-1 DNA and **(D)** HIV-1 RNA in tissues at single time point were listed with each dot representing an individual animal. Values below the horizontal line indicated that viral DNA and RNA were below the limit of detection.

HIV-1 infection is established in target tissues before viremia can be detected (23). To determine the early distribution of HIV-1 infection in tissues, gut, spleen, lung, liver, brain, and kidney were procured then evaluated after animal sacrifices (Figures 1A, 2A). Tissue HIV-1 DNA and RNA were quantified by ultrasensitive semi-nested real-time qPCR (19). In general, tissue viral levels were higher in longer infected hu-HSC and hu-PBL mice. In addition, tissue viral DNA and RNA corresponded to what was detected in plasma in both animal models (Figures 4A,B, Figures 5A,B).

In hu-HSC mice, HIV-1 DNA, and RNA were detected at low levels within 3 days after viral challenge, from spleen, lung, and liver in 1/5 animals (Figures 4C,D). The same tissues examined at days 5 and 7 showed infection in 2/5 animals while HIV-1 remained undetected in other tested tissues. In the animals infected for 14 days, viral DNA, and RNA were observed in 3/5 gut, spleen, lung, and kidney tissues, and 2/5 liver samples (Figures 4C,D). However, HIV-1 was not detected in hu-HSC mouse brain until day 28. At 28 and 42 days, virus was readily observed throughout all tested tissues from all infected animals (Figures 4C,D).

HIV-1 was detected earlier and at higher levels in hu-PBL vs. hu-HSC mouse tissues at all-time points (Figures 5C,D). Viral DNA and RNA were seen by day 3 in 81% (29/36) gut, spleen, lung, liver, brain, and kidney tissues examined. At day 5, 93% (28/30) infected tissue were HIV nucleic acid positive. Notably, 67% (6/9) brain tissue samples from days 3 and 5 showed absent virus that supported later seeding for this tissue compartment. In the animals infected for 7 and 14 days virus was readily observed in all tissues (Figures 5C,D). HIV-1 DNA levels in gut were higher than that in all other tissues and supported the notion that gut serves as a prominent virus tissue compartment (Figure 5A). Altogether, these data suggested that both peripheral and tissue HIV-1 compartments were rapidly established in hu-HSC (day-14) and hu-PBL (day-3) mouse models, but much faster in hu-PBL than in hu-HSC mice.

### Confirmatory Tests of Viral Gene Expression in Infected Humanized Mice

**Figure 6:**
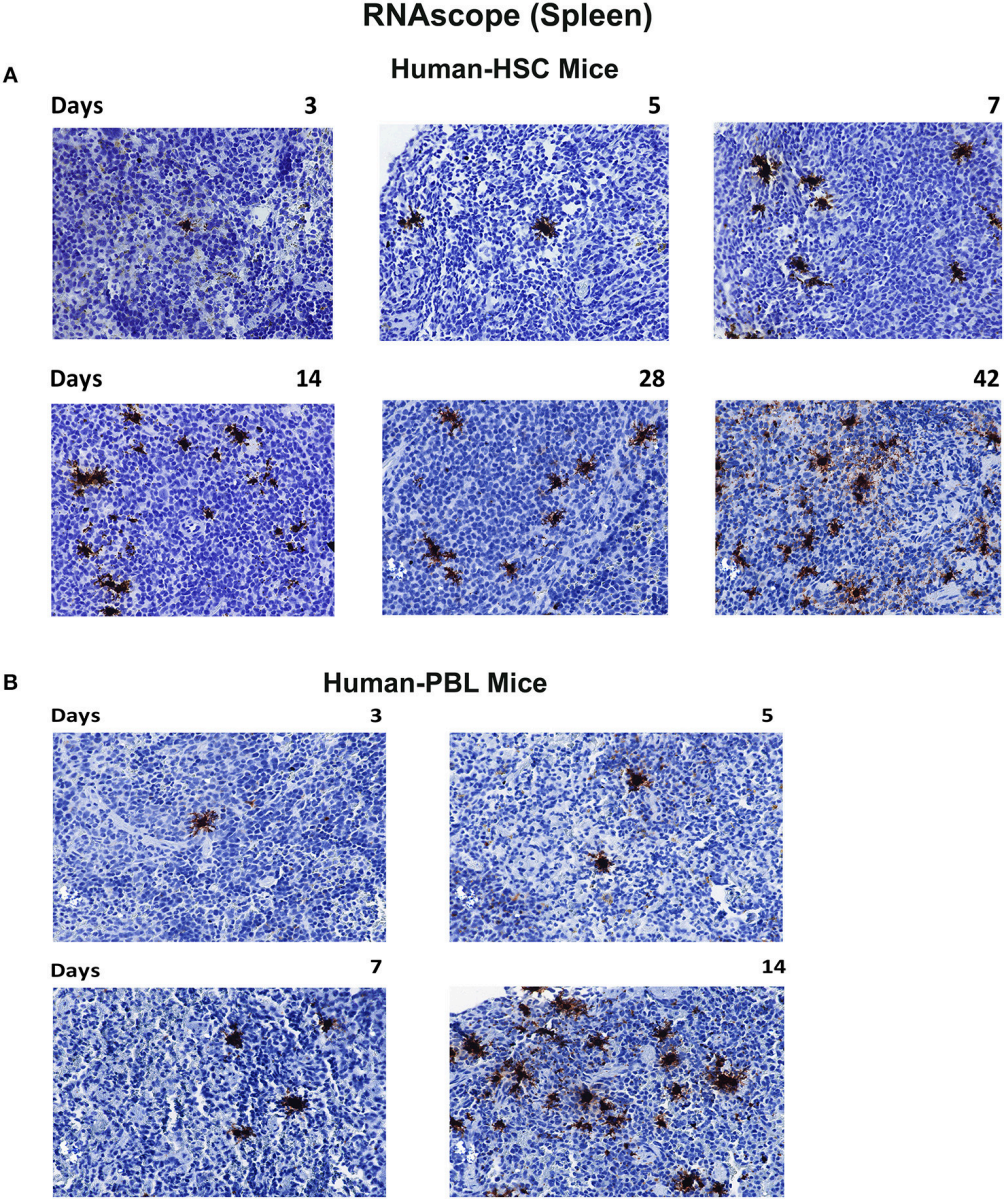
Viral RNA tissue expression in infected humanized mice. Spleen tissues of hu-HSC **(A)** and hu-PBL **(B)** mice were collected with formalin fixed and paraffin embedded at necropsy. Five micrometer thick slices were prepared for RNAscope assays. Representative images from each group were shown with HIV-1 RNA labeled as single or cluster of brown dots. Images were taken at 40 x objective magnifications.

To confirm tissue compartmentalization in early HIV-1 infected humanized mice, spleen sections were obtained then tested by RNAscope that can detect up to 1–2 copies of viral RNA (representative images shown in Figure 6). An antisense HIV-1 Clade B probe was employed which covers nearly entire viral genome (except LTR region). To this end, spleen HIV-1 RNA was shown as a single or cluster of brown dots, at the earliest stage of infection in both mouse models. In hu-HSC mice, HIV-1 RNA was visualized within 3 days of infection, which reaffirmed the rapid set-up of viral tissue compartment. As infection proceeded, virus spread as shown in multiple clusters of brown dots within each tissue section. By day 42, viral burden was much more prominent with invaded cells aggregated throughout the observed field of interest (Figure 6A). In hu-PBL mice, HIV-1 RNA was observed in all infected animal spleens at each time point. Viral RNA levels were comparable or higher in hu-PBL than hu-HSC mice at equivalent time courses (Figure 6B).

### Viral Protein Expression in Infected Humanized Mice

**Figure 7:**
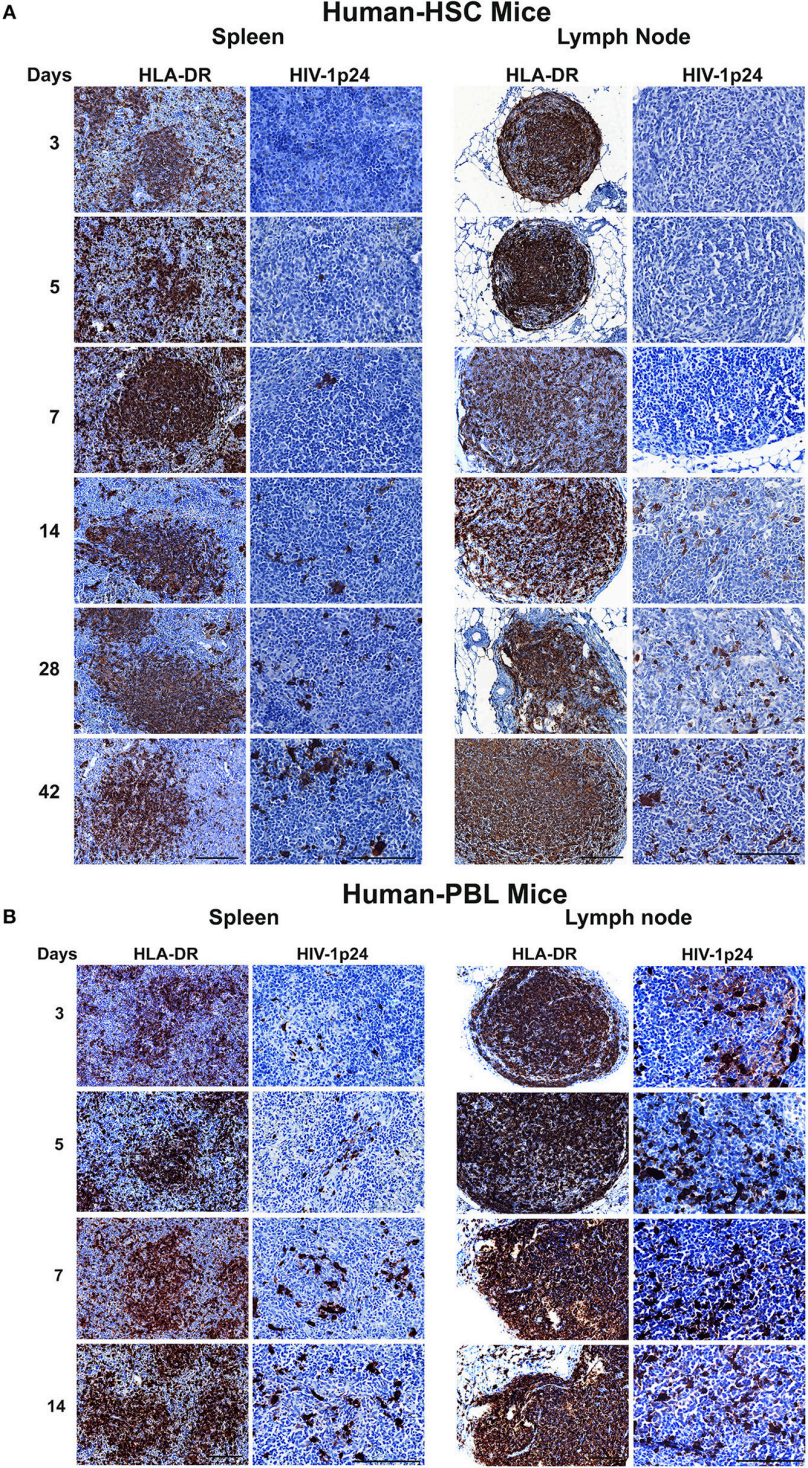
HIV-1p24 expression in infected humanized mice. Spleen and lymph node samples were collected from **(A)** hu-HSC and **(B)** hu-PBL mice at necropsy then formalin fixed and paraffin embedded. Five micrometer thick sections were cut then stained with human HLA-DR and HIV-1p24 antibodies. Representative images from each group were selected and pictures were captured for both markers (shown as brown dots) from individual animals. Human HLA-DR images were taken at 20 x objective magnifications and HIV-1p24 images were captured at 40 x objective magnifications.

HIV-1p24 is a capsid component that is among the earliest expressed viral proteins. To assess its presence in infected tissues we employed immunohistochemistry assays to trace HIV-1p24 along with human HLA-DR staining. Representative photomicrographs were taken from each tissue sample stained with both antibodies (Figure 7). In hu-HSC mice, while HLA-DR+ cells were easily seen in the observation field, HIV-1p24 cells, however, were observed only in 1/5 animal spleens infected for 5 or 7 days. No infected cells were seen at 3 days. By day 14, 3/5 animals were HIV-1p24 positive. These three animals were the same ones where virus was detected by viral qPCR and RNAscope tests (Figures 4, **6**). At 28 and 42 days, HIV-1p24 stained cells were demonstrated in all infected animals (Figure 7A). In hu-HSC lymph nodes, HIV-1p24 antigens were detected starting at 14 days after infection and increased over time (Figure 7A).

In hu-PBL mice, HIV-1p24 antigens were captured in all infected animal spleens and lymph nodes during serial necropsies while human HLA-DR+ cells were well reconstituted (Figure 7B). Levels of HIV-1 p24 and nucleic acids in spleen measured by immunostaining and qPCR and RNAscope tests paralleled one another (Figures 5, 6B). During the equivalent infection windows, tissue HIV-1p24 expansion was more aggressive in hu-PBL than that in hu-HSC mice (Figure 7B). These data together confirmed that the quickly established HIV-1 infection in both models were replication-competent and virus spread more aggressively in hu-PBL than in hu-HSC mice.

### Host Immunity in Humanized Mouse Models

**Figure 8:**
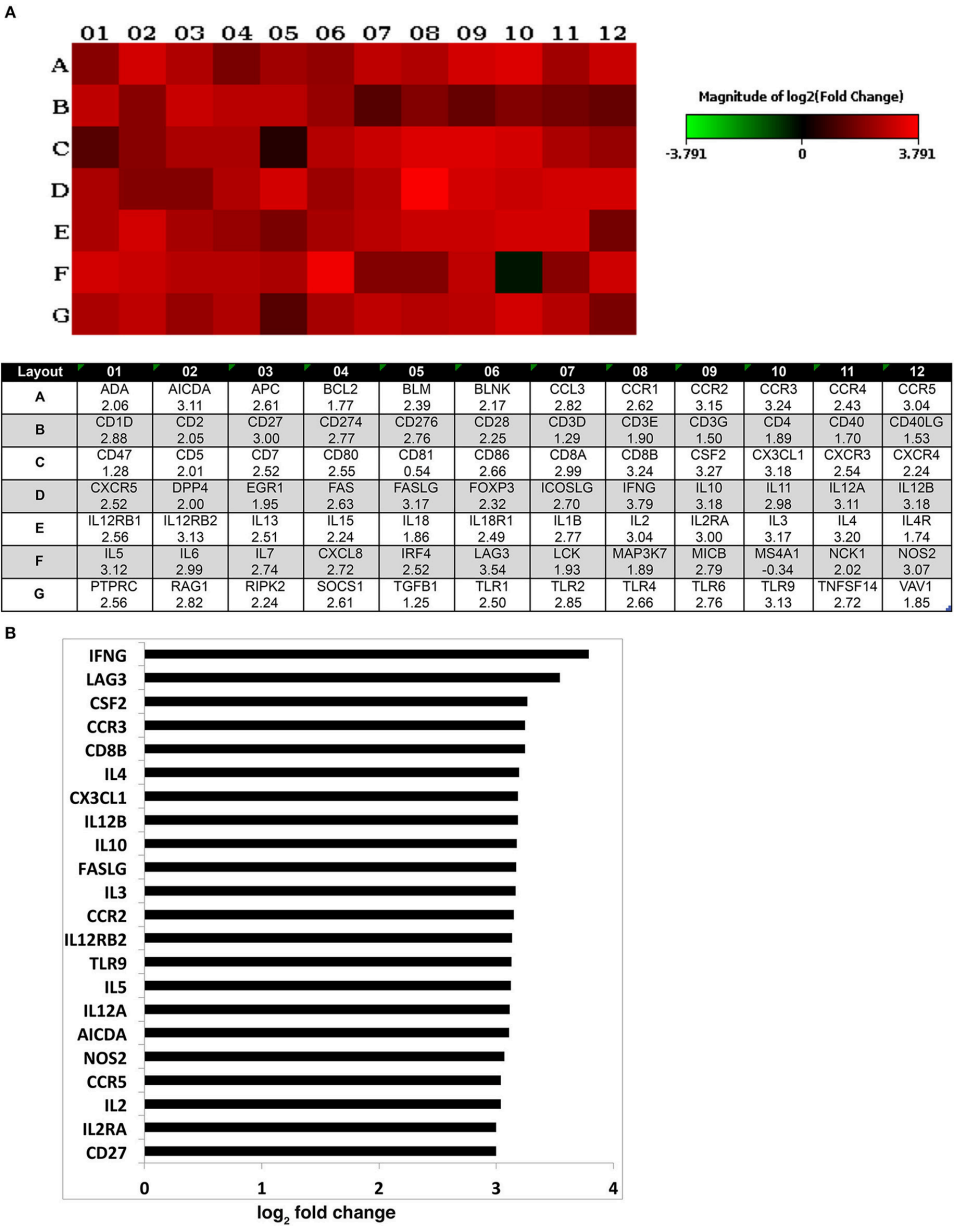
Expression of human immune activation markers in humanized mice. Total RNA was isolated from uninfected humanized mouse spleens and analyzed for the expression of markers for human immune activation. A total of 84 genes were evaluated and compared between the two animal models. **(A)** Heatmap depicted the differentially expressed genes (for 1–12) associated with immune activation in hu- PBL compared against HSC mice (for A–G). The log_2_ fold change of the individual gene is listed in the bottom panel. **(B)** Differentially expressed genes with log_2_ fold change of ≥3 are outlined that are expressed in hu-PBL spleens over what was found in HSC mice. A complete gene list can be found in Table S2.

**Figure 9:**
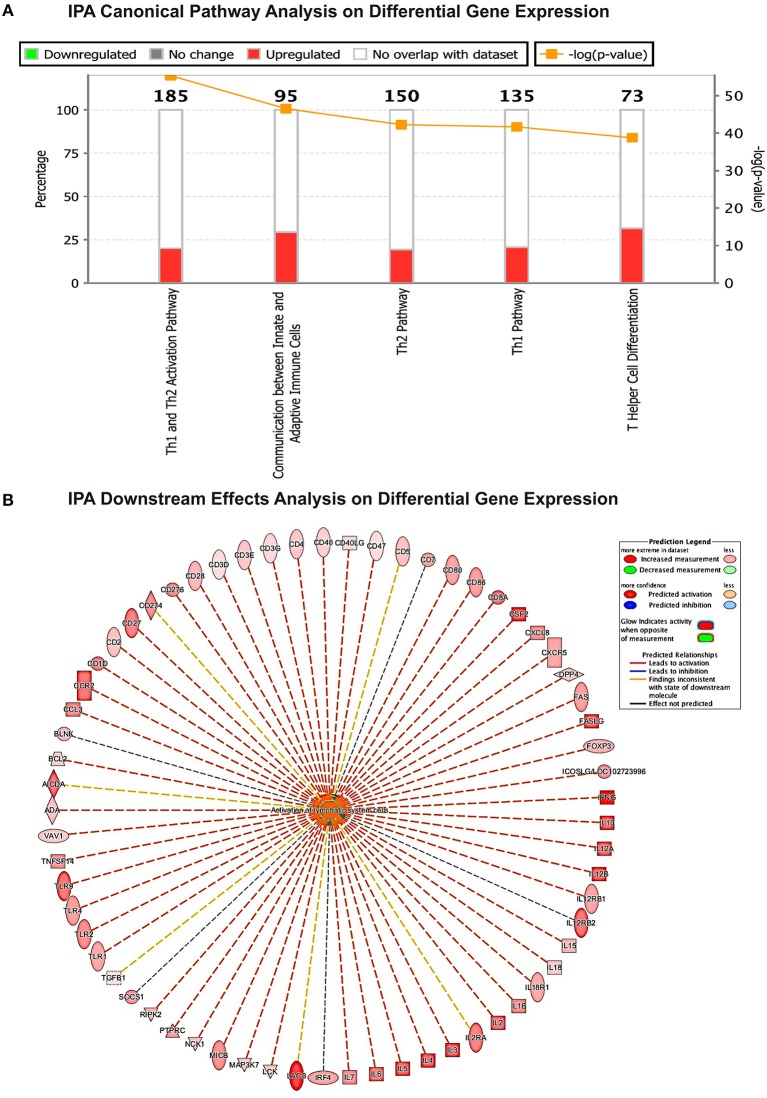
Gene expression patterns analysis by IPA in humanized mice. **(A)** Canonical pathway gene analyses. The stacked bar chart demonstrates the percentage of upregulated (red) and downregulated (green), as well as non-overlapped (white) genes from the prestored genebank in IPA (numbers listed at the top of each bar). The right y-axis displays the –log of *p*-Value calculated by Fisher's Exact Test illustrates the significance of each canonical pathway. **(B)** Downstream biological effects prediction. The most relevant downstream effect predicted by IPA was lymphoid activation. A set of 65 genes that were differentially expressed between hu-PBL and hu-HSC mice co-regulate this pathway. The putative function was located in the center while the related regulator listed at the periphery. The type of interaction is indicated by red (prediction of activation), blue (prediction of inhibition), yellow (inconsistent), and gray (related, not predicted).

Different strategies of humanization shape unique cellular integrations in humanized mice. Previous studies observed that the engrafted human T cells in hu-PBL mice expressed a predominated memory/activated (CD45RO) phenotype that supports HIV-1 infection (24) while in hu-HSC mice approximately 50% of human T cells are naive (CD45RA) that are less susceptible to HIV-1 infection (19). Therefore, viral infection is usually more aggressive in hu-PBL mice than that in hu-HSC mice (10). In the current study, we also observed a similar pattern during early HIV-1 infection where virus was seeded at accelerated rates in hu- PBL than in HSC mice. To further characterize and compare the intrinsic host environment in both mouse models that may affect HIV-1 infection, we adopted naïve hu-HSC and hu-PBL mice (*n* = 3/group) with comparable human cell reconstitutions (Table S1). In these animals, immune-linked host gene expression was tested. Total RNA was isolated from individual animal spleen and a total of 84 gene expressions were evaluated. Overall, increases in gene expressions paralleled adaptive immune activation and were most prominent in hu- PBL vs. HSC mice (Figure 8 and Table S2). Upregulated T cell genes were readily observed affecting cell activation (e.g. CD2, CD3, CD4, CD8, FOXP3, and LAG3), proliferation (e.g. CD28, IL2, IL1β, IL18, and TNFSF14), and differentiation (e.g. CD27, CD80, CD86, and IL15). Two major co-receptors for HIV-1 entry, CCR5, and CXCR4, were also found to be upregulated in hu-PBL compared to HSC mice. The elevated B cell activation and proliferation markers included CD27, CD40, CD80, CD81, IL2, and IL10. To investigate how these differentially expressed molecules may impact the host environment, we subjected the genes with fold changes above 2 (81/84) to Ingenuity Pathway Analysis (IPA). These tests revealed that the top canonical pathways affected in hu-PBL over HSC mice were (1) Th1 and Th2 activation pathway (*p* = 4.62E-56); (2) innate and adaptive immunocyte communications (*p* = 3.03E-47); (3) Th2 (*p* = 5.58E-43); (4) Th1 (*p* = 2.14E-42) and (5) T-helper cell differentiation (*p* = 1.76E-39, Figure 9A). All five pathways are engaged in T cell regulation. Downstream Effects Analysis was performed to assess regulatory hierarchy. A total of 500 gene-related diseases or functions each with a minimum of 10 molecules related were predicted and top 10 functions were listed (Table S3). The differential genetic network in the hu-PBL mice was most significantly correlated with the activation of lymphatic systems with 80% (65/81) of the input genes involved and 83% (54/65) led to systemic activation responses (Figure 9B). A spectrum of inflammation-associated genes was also upregulated in hu-PBL as compared to hu-HSC mice, including both pro-inflammatory (e.g., IL1, IL17, IFN γ, TNFα, CXCL3, and CXCL8) and anti-inflammatory (e.g., IL4, IL6, IL10, IL12, IL13, and TNFβ) molecules (Figure 8 and Table S2). IPA analysis confirmed that this genetic pattern was associated with inflammatory responses with 67% (54/81) of the input genes involved and 81% (44/54) linked to cell activation pathways (Table S3 and Figure S2). Taken together, these data support the notion that an established immune activated and inflammatory tissue environment facilitates HIV-1 infection and dissemination.

## Discussion

Early ART intervention restricts the HIV-1 reservoir size (25–27) and may achieve long-term viral remission in select infected individuals (28, 29). However, all patients inevitably experience viral relapse even when treatment is started as early as 14 days after infection (6). It is thus important to determine viral compartmentalization in cells and tissues. Nonetheless, it is not possible to accurately answer this question in an infected human. Therefore, in the current study we traced HIV-1 peripheral and tissue dissemination after infection in humanized mice used to reflect the temporal dynamics of tissue infection. The major advancements of this study were direct comparisons between hu- HSC and PBL mice using a dual tropic HIV-1 strain (HIV-1_ADA_) in study (30). Multiple time points after HIV-1 infection to reflect a complete picture of early viral dynamics as well as host immune responses. By comparing the viral-host kinetics, we were able to identify the host factors that affect early events of HIV-1 infection. Limitations in accessing human samples to correlate immune responses can be achieved through the use of humanized mice.

Herein, two well-studied chimeric humanized mouse models were used in this report with divergent biologic and immune characteristics. Hu-HSC mice were made after engrafting human CD34+ HSC into new born NSG mice (31). After cell differentiation and maturation, mice are reconstituted with multiple lineages of human immune cells. The cellular type and composition in hu-HSC mice are more similar to human (T and B cells and monocyte-macrophages) with a life expectancy of more than a year. Hu-PBL mice are produced by implanting human peripheral blood lymphocytes into the adult NSG mice. This leads to dominant human lymphocyte reconstitution of up to 95% T cells within 2–3 days (32). However, as a result of GvHD, the life span of the viral immune responses in these animals can only be measured for a single month. Results from both models allowed us not only able to trace early HIV-1 infection, but investigate how host environments may affect viral-host outcomes (33). Peritoneal infections were performed to ensure reproducibility between animals.

Effector memory CD4+ T cells are the primary targets of HIV-1 and their depletion parallels the development of the acquired immune deficiency syndrome (23). CD4+ T cell loss is observed within months of HIV-1 infection from both peripheral blood and lymphoid tissues (34). In the current study, we observed modest CD4+ T depletion in blood of hu-HSC mice as early as 3 days after infection that progressed over time. A similar trend was also observed in infected splenocytes and bone marrow. Altogether, such results indicate that human immune function is impaired at the earliest stage of infection (35). CD8+ T cell percentages were elevated in parallel to CD4+ T cell losses. In hu-PBL mice, peripheral CD4+ T cell depletion was more significant than what was observed in hu-HSC mice. This reflected a highly activated cell phenotype facilitating productive HIV-1 infection and cellular degradation.

Plasma HIV-1 RNA is first seen within 3 weeks after HIV-1 infection in humans (36, 37). Due to the difficulty of early HIV-1 screen in the clinic, a gap between initial viral exposure, and estimated infection period is inevitable, which can be bridged using suitable animal models. In the current study, peripheral viral load was detected in 40% (2/5) of hu-HSC animals by days 3, 5, and 7 after infection each and 100% by day 14. It implied that peripheral viral replication might be established earlier than what is usually observed in humans given the possibility that highly sensitive techniques may further improve detection limit. In hu-PBL mice peripheral viral load was fully expressed in all monitored animals within 3 days of infection with a peak viremia seen at day 7. The data clearly support the notion that viral replication is linked to immune activation.

Tissue HIV-1 DNA and RNA was first detected by semi-nested qPCR at 3 days after infection in hu-HSC mouse spleen, lung, and liver, demonstrating that infection was established and disseminated to multiple tissues rapidly while plasma viral load is extremely low or undetectable. Interestingly, at 5 and 7 days after infection, viral nuclear acids were recovered from each of these tissues in 2/5 animals and supported the fact that all three tissue compartments were seeded by virus at the earliest stage of infection. The observation of HIV-1 RNA and HIV-1p24 antigen on spleen sections supported that it could serve as a major anatomical infectious site (38). In addition, infected cells were highly enriched within the lymphoid follicles. This is likely due to known higher numbers of reconstituted human cells in follicular regions and indicates that this lymphoid subregion may play a major role during early establishment of tissue infections. As the germinal centers are poorly developed in humanized mice, their role in viral compartmentalization, and as potential reservoirs for infection requires further research if a potential viral sanctuary can be realized and most notably during effective ART (39). At 14 days after HIV-1 infection, virus was easily seen in spleen, lung, liver, gut, and kidney. Tissue viral DNA and RNA levels were dramatically increased compared to the early time points, accordant with plasma HIV-1 RNA. One can speculate that multiple peripheral and tissue sanctuaries have been established at early points after viral infection, which may illustrate the hurdles that need be overcome to achieve viral eradication (6). These observations highlight hu-HSC mice as a robust model for studying the earlier stages of HIV-1 infection and support our own prior works using the model to investigate viral cellular and tissue replication patterns during chronic infections (19). Indeed in hu-HSC mice, HIV-1 replication is readily identified in a broad range of bone marrow, spleen, lung, gut, brain, kidney, and liver tissues as well as CD34+ progenitors, monocyte-macrophages, dendritic cells, and CD4+ stem cell memory, naïve memory, central memory, effector memory, and regulatory T cells. All were identified in animals after 5 to 14 weeks of viral infection.

Lymph nodes are major tissue compartments that harbor HIV-1 (40). In the current study, we did not detect HIV-1p24 in hu-HSC mouse lymph nodes until 14 days after infection. This reflected, in measure, the underdeveloped lymph nodes in immune deficient animals (41). While gut-associated lymphoid tissue or GALT is one of the earliest observed infected tissue during acute HIV-1 infection (42) we also were not able to detect HIV-1 infection in hu-HSC mouse GALT until day 14. This was later than what was observed from spleen, lung, and liver. These data reflect the relatively low humanization operative in hu-HSC mouse GALT (43). This limitation restricts studies of viral transmission (44). However, even considering the limitations of the model infection of GALT showed high levels of infection at later time points. The low reconstitution of human immune cells may also explain delayed HIV-1 infection in hu-HSC mouse brains. A recent study reported that within early infected individuals (median 15 days), HIV-1 RNA was observed in cerebrospinal fluid from 83%, 15/18 infected subjects, with the earliest detection by 8 days (37). However, according to variant humanized mouse models studied, HIV-1 seeding in the central neural system was generally much more delayed (16, 19, 45). This discordance demonstrates some of the limitation of our current humanized mouse models. Of interest to the current studies are our prior experiences in using hu-HSC mice that showed sustained bone marrow viral burden (19, 46). Although bone marrow HIV-1 infection was not measured in the current study, the progressive decline of CD4+ T cells indicated that active viral replication was rapidly established in the hu-HSC mouse bone marrow. Notably and distinct from hu-HSC mice, HIV-1 was seeded into tissue compartments more rapidly in hu-PBL mice. At 3 days post-infection, HIV-1 DNA and RNA were readily detected across a wide range of tested tissues, including brains. HIV-1p24 was also observed in the 3-day infected lymph node sections. Viral levels were higher in hu-PBL mice at the same time courses compared to that in hu-HSC mice. Altogether, these results strengthen the notion that the host microenvironment is closely linked to early HIV-1 replication dynamics.

Previous studies by others showed that low levels of T cell activation and proliferation lead to reduced HIV-1 susceptibility (47, 48). Nevertheless, comorbid factors such as sexually transmitted infectious diseases substantially increased the risks of HIV-1 acquisition and transmission as well as affecting viral load. All are known to be associated with inflammation and immune activation (49–51). Our data support the idea that immune activation markers predict viral susceptibility in mouse models of human disease. Comparisons in host tissue environments were made at the transcriptional level in tissues from both models. After identifying viral dynamics after HIV-1 infection of hu-PBL mice, immune-activated genes linked to T and B cells were upregulated when compared to hu-HSC mice. These data support the idea that immune activation that occurs prior to infection could predict early HIV-1 infection dynamics in these animal models. In addition, a wide range of inflammation-associated genes was upregulated in the hu- PBL compared to HSC mice. While pro-inflammatory conditions facilitate viral acquisition, and promote T cell activation (52) anti-inflammatory factors serve to maintain systemic homeostasis. Noteworthy, a recent report demonstrated that a systemic proinflammatory signature was established by as early as 24 h after SIV infection of rhesus macaques (9). It will be interesting to evaluate how early inflammasome activated in HIV-1 infection affects early viral dynamics. We posit that immune-activation and inflammation explains early HIV-1 infection, rapid viral dissemination, and accelerated CD4+ T cell loss in the hu-HSC mice.

Recently we demonstrated that hu-HSC mice infected with HIV-1_NL4−3_ strain (53) expressed high levels of HIV-1 replication in peripheral blood, gut, spleen, lung, liver, brain, kidney, lymph node, and bone marrow. These results illustrated that viral factors also affect the formation of HIV-1 infection in tissue compartments. In addition, a recent report found that after acute intravaginal challenge of HIV-1_BaL_ on humanized Rag1KO.IL2RγcKO.NOD mice expressing HLA class II (DR4) molecule (DRAG) mice (16), virus was detected at certain tissues by day 4 while brain was lastly infected until day 21. This also suggested that different infection routes and genetic background should be considered in reflecting what would be operative in an infected human host. While we understand that the intraperitoneal route used to establish viral infection does not reflect what is operative during natural conditions. However, we performed this route to ensure infection was operative in 100% of challenged animals and was able to explore viral compartmentalization during the evolution of persistent viral infection. While early HIV-1 infection remains challenging to identify and investigate in the clinic, humanized mouse models allow researchers to determine how, where and at what levels virus gains a foot hold in tissue sites and prior to any or all therapeutic or cure strategies. Indeed, based on this work, our group has shown that combinations of long acting slow effective release antiretroviral therapy (LASER ART) and CRISPR-Cas9 for viral excision can lead to permanent HIV-1 elimination. In up to one third of infected humanized mice (our unpublished observations) and supports the use of this model in viral eradication schemes.

In conclusion, by using humanized mouse models, our study identified a wide range of tissue compartments, and their temporal and spatial dynamics during early HIV-1 infection. The four major findings from this study are summarized as *First*, HIV-1 infection was identified in multiple tissue compartments as early as 3 days post-infection using highly sensitive detection techniques in two different humanized animal models. Second HIV-1 was detected in all tissue by day 14 in hu-HSC mice. *Third*, tissue viral replication patterns were linked to markers of immune activation and immunity for each animal model that included T cell maturation and inflammation. *Fourth*, spleen, lung, and liver were among the earliest infected tissues and sustained heavy viral burden throughout the monitoring period as shown in proviral DNA amplifications. It is noteworthy that the tissue types listed above do not cover all the human anatomical viral sanctuaries. Others tissues that require analyses in humanized mice include but not limited by thymus, male and female reproductive tract, skin, and adipose tissue (54, 55). Even accepting the limitation of both models and underdevelopment of secondary lymphoid tissues this information will instruct us on the guideline of early ART intervention and development of tissue-specific ART. It has been an intriguing question that whether a window exists for “HIV-1 cure,” if ART is administrated soon after viral exposure to maximize the restriction of viral replication followed by combinational strategies targeting the residual proviral DNA. Using humanized mouse model under controlled conditions, we will be able to answer this question, which will benefit the translation of clinical investigation.

## Author Contributions

PD and HG conceived and designed the experiments and interpreted the data sets. PD and HS performed the virologic, immunologic, molecular biology and transcriptomic experiments, and interpreted and plotted the datasets. Both prepared the figures for publication and wrote the manuscript. YC, SS, and SM generated the mice used in the study and performed immunologic testing. Manuscript editing was performed by HG with the assistance of PD, SG, and LP.

### Conflict of Interest Statement

The authors declare that the research was conducted in the absence of any commercial or financial relationships that could be construed as a potential conflict of interest.
