# Supplementary material for: Immune Activations and Viral Tissue Compartmentalization During Progressive HIV-1 Infection of Humanized Mice
Source: Front Immunol. 2019 Feb 28;10:340. doi: 10.3389/fimmu.2019.00340 (PMC6403174; doi:10.3389/fimmu.2019.00340)
Supplement: Supplementary file 1 [file Data_Sheet_1.PDF]

Immune Activation Defines Viral Tissue Compartmentalization During Progressive HIV-1 Infection of Humanized Mice

SUPPLEMENTARY MATERIALS

Hang Su[a], Yan Cheng[a], Sruthi Sravanam[a], Saumi Mathews[a], Santhi Gorantla[a], Larisa Y. Poluektova[a], Prasanta K. Dash[a, *] and Howard E. Gendelman[a, b, * ‡]

[a]*Department of Pharmacology and Experimental Neuroscience, College of Medicine, University of Nebraska Medical Center, Omaha, NE, USA*

[b]*Department of Pharmaceutical Sciences, College of Pharmacy, University of Nebraska Medical Center, Omaha, NE, USA*

Running Title: Progressive HIV-1-infection of humanized mice

***Corresponding authors:** Prasanta K. Dash, Ph.D., email: pdash@unmc.edu; phone: 402-559-8925; and Howard E. Gendelman, M.D., email: hegendel@unmc.edu; phone: 402-559-8920

‡**Communication author** (for submission and review): Howard E. Gendelman, M.D., Department of Pharmacology and Experimental Neuroscience, University of Nebraska Medical Center, Omaha, NE, USA; phone: 402-559-8920; fax: 402-559-3744; email: hegendel@unmc.edu

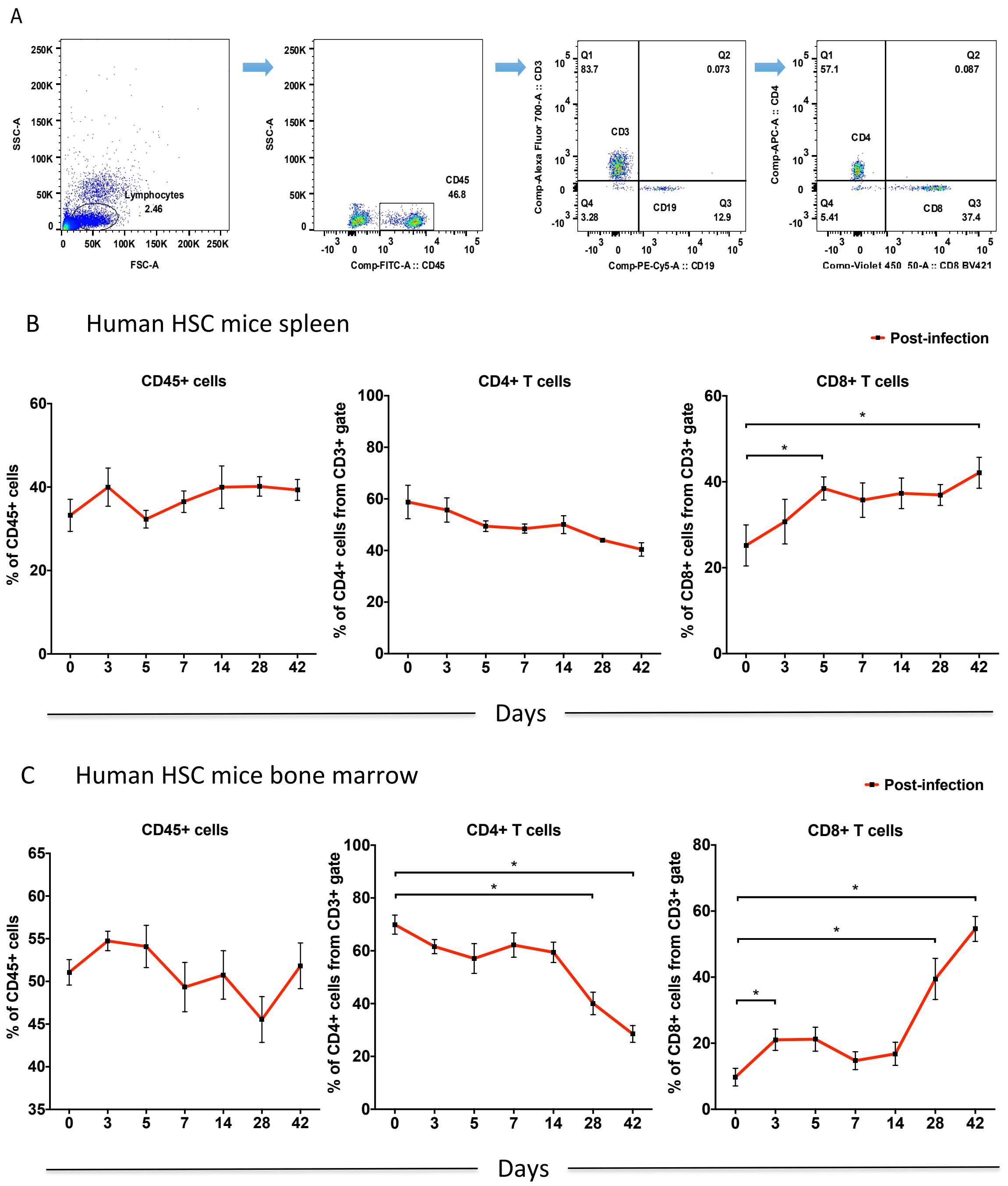

Figure S1. Human cells in spleen and bone marrow in infected hu-HSC mice. (**A**) The gating strategy for this study is shown. In brief, human cells (hCD45+) were gated from total lymphocytes and subsequently seperated into human T and B (hCD3+ and hCD19+) lymphocytes. Human CD4+ and CD8+ T cells were gated from hCD3+ cell populations. (**B**) Splenocytes and (**C**) bone marrow cells were acquired from hu-HSC mice at serial time points (days 0, 3, 5, 7, 14, 28, and 42) after HIV-1 infection and subjected to flow cytometric analyses. Numbers of human CD45+, CD4+, and CD8+ cells in both tissues are expressed as mean ± SEM. Values were statistically significant if $^{*}p<0.05$.

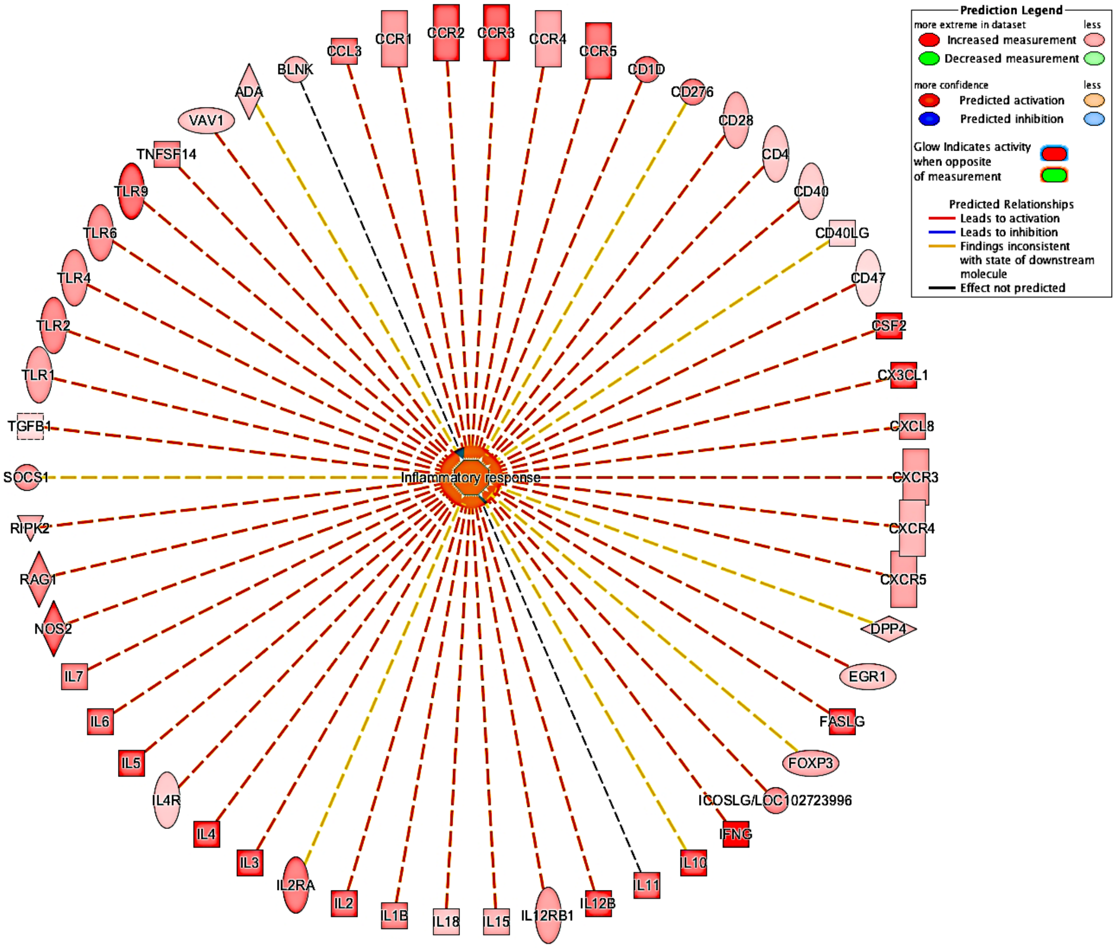

Figure S2. Altered expression of humanized mouse inflammatory genes by IPA. Total spleen RNA was isolated from hu-HSC and hu-PBL mice and probed using RT^2 Profiler PCR Array for T & B cell activation profiles. Hu-HSC mice were chosen as controls while hu-PBL mice were the test group. The fold changes of human-specific genetic expression (hu-PBL/hu-HSC) were determined by SABioscience RT^2 Profiler PCR Array Data Analysis software, version 3.5. IPA. This program was applied for predicting biological functions based on differentially expressed genes acquired in the dataset. Inflammatory gene markers were the most significantly affected pathways (from a total of 54 genes) differentially expressed between hu-PBL and hu-HSC mice. The putative function was located in the center while the related regulators listed. The type of interaction is indicated by red (prediction of activation), blue (prediction of inhibition), yellow (findings inconsistent), and gray (known to be related, but not predicted).

Table S1. Human cell engraftment in individual humanized mice (for IPA analyses)

Peripheral human cells	Humanized mouse model					
	Hu-HSC (mouse #)			Hu-PBL (mouse #)		
	3631	3641	3655	A1	A2	A3
**% CD45**	45.6	63.3	53.8	52.2	49.1	70.2
**% CD3**	70.9	66.4	85.9	97.1	96.3	96.9
**% CD4**	70.3	71.1	75.8	49.8	43.6	32.2
**% CD8**	24.0	22.5	21.2	38.5	40.3	53.3

Table S2. Changes of human gene expressions in hu- PBL versus HSC mice

Gene Symbol	Gene Description	Fold Change
IFNG	Interferon, gamma	13.85
LAG3	Lymphocyte-activation gene 3	11.67
CSF2	Colony stimulating factor 2 (granulocyte-macrophage)	9.63
CD8B	CD8b molecule	9.48
CCR3	Chemokine (C-C motif) receptor 3	9.48
IL4	Interleukin 4	9.16
IL12B	Interleukin 12B (natural killer cell stimulatory factor 2)	9.09
CX3CL1	Chemokine (C-X3-C motif) ligand 1	9.09
IL10	Interleukin 10	9.05
FASLG	Fas ligand (TNF superfamily, member 6)	9.01
IL3	Interleukin 3 (colony-stimulating factor, multiple)	8.97
CCR2	Chemokine (C-C motif) receptor 2	8.88
IL12RB2	Interleukin 12 receptor, beta 2	8.78
TLR9	Toll-like receptor 9	8.76
IL5	Interleukin 5 (colony-stimulating factor, eosinophil)	8.72
IL12A	Interleukin 12A (natural killer cell stimulatory factor 1)	8.66
AICDA	Activation-induced cytidine deaminase	8.62
NOS2	Nitric oxide synthase 2, inducible	8.41
CCR5	Chemokine (C-C motif) receptor 5	8.23
IL2	Interleukin 2	8.21
IL2RA	Interleukin 2 receptor, alpha	8.01
CD27	CD27 molecule	7.99
IL6	Interleukin 6 (interferon, beta 2)	7.93
CD8A	CD8a molecule	7.93
IL11	Interleukin 11	7.9
CD1D	CD1d molecule	7.35
TLR2	Toll-like receptor 2	7.2
CCL3	Chemokine (C-C motif) ligand 3	7.08
RAG1	Recombination activating gene 1	7.07
MICB	MHC class I polypeptide-related sequence B	6.94
IL1B	Interleukin 1, beta	6.84
CD274	CD274 molecule	6.8
CD276	CD276 molecule	6.78
TLR6	Toll-like receptor 6	6.76

IL7	Interleukin 7	6.7
CXCL8	Interleukin 8	6.61
TNFSF14	Tumor necrosis factor (ligand) superfamily, member 14	6.58
ICOSLG	Inducible T-cell co-stimulator ligand	6.5
TLR4	Toll-like receptor 4	6.31
CD86	CD86 molecule	6.3
FAS	Fas (TNF receptor superfamily, member 6)	6.2
CCR1	Chemokine (C-C motif) receptor 1	6.14
SOCS1	Suppressor of cytokine signaling 1	6.12
APC	Adenomatous polyposis coli	6.12
PTPRC	Protein tyrosine phosphatase, receptor type, C	5.89
IL12RB1	Interleukin 12 receptor, beta 1	5.89
CD80	CD80 molecule	5.85
CXCR3	Chemokine (C-X-C motif) receptor 3	5.83
CXCR5	Chemokine (C-X-C motif) receptor 5	5.75
CD7	CD7 molecule	5.75
IRF4	Interferon regulatory factor 4	5.74
IL13	Interleukin 13	5.68
TLR1	Toll-like receptor 1	5.65
IL18R1	Interleukin 18 receptor 1	5.62
CCR4	Chemokine (C-C motif) receptor 4	5.39
BLM	Bloom syndrome, RecQ helicase-like	5.23
FOXP3	Forkhead box P3	4.99
CD28	CD28 molecule	4.76
RIPK2	Receptor-interacting serine-threonine kinase 2	4.73
CXCR4	Chemokine (C-X-C motif) receptor 4	4.73
IL15	Interleukin 15	4.71
BLNK	B-cell linker	4.51
ADA	Adenosine deaminase	4.17
CD2	CD2 molecule	4.13
NCK1	NCK adaptor protein 1	4.05
CD5	CD5 molecule	4.03
DPP4	Dipeptidyl-peptidase 4	3.99
EGR1	Early growth response 1	3.86
LCK	Lymphocyte-specific protein tyrosine kinase	3.81
CD3E	CD3e molecule, epsilon (CD3-TCR complex)	3.72
MAP3K7	Mitogen-activated protein kinase kinase kinase 7	3.71
CD4	CD4 molecule	3.71
IL18	Interleukin 18 (interferon-gamma-inducing factor)	3.62
VAV1	Vav 1 guanine nucleotide exchange factor	3.61

BCL2	B-cell CLL/lymphoma 2	3.42
IL4R	Interleukin 4 receptor	3.34
CD40	CD40 molecule, TNF receptor superfamily member 5	3.24
CD40LG	CD40 ligand	2.89
CD3G	CD3g molecule, gamma (CD3-TCR complex)	2.83
CD3D	CD3d molecule, delta (CD3-TCR complex)	2.44
CD47	CD47 molecule	2.43
TGFB1	Transforming growth factor, beta 1	2.38
CD81	CD81 molecule	1.45
MS4A1	Membrane-spanning 4-domains, subfamily A, member 1	-1.27

Table S3. p-value ranking of top 10 IPA assayed categories and function observed by IPA in hu- PBL and HSC mice

Categories	Functions	p-Value	Activation z-score	Molecule numbers
Cell-To-Cell Signaling and Interaction	Activation of lymphatic system cells	4.22E-86	6.324	65
Cell-To-Cell Signaling and Interaction, Hematological System Development and Function, Immune Cell Trafficking, Inflammatory Response	Activation of mononuclear leukocytes	3.94E-85	6.308	65
Cell-To-Cell Signaling and Interaction, Hematological System Development and Function, Immune Cell Trafficking, Inflammatory Response	Activation of lymphocytes	1.67E-84	6.255	64
Cell-To-Cell Signaling and Interaction, Hematological System Development and Function, Immune Cell Trafficking, Inflammatory Response	Activation of leukocytes	1.46E-83	6.523	70
Cell-To-Cell Signaling and Interaction	Activation of cells	8.43E-76	6.837	72
Hematological System Development and Function, Tissue Morphology	Quantity of mononuclear leukocytes	4.43E-74	4.681	67
Cellular Development, Cellular Growth and Proliferation, Hematological System Development and Function, Hematopoiesis, Lymphoid Tissue Structure and Development	Differentiation of mononuclear leukocytes	1.48E-73	7.253	66
Hematological System Development and Function, Tissue Morphology	Quantity of leukocytes	2.38E-73	4.435	70

Cellular Development, Cellular Growth and Proliferation, Hematological System Development and Function, Hematopoiesis, Lymphoid Tissue Structure and Development	Leukopoiesis	3.59E-73	7.542	68
Cellular Development, Cellular Growth and Proliferation, Hematological System Development and Function, Hematopoiesis, Lymphoid Tissue Structure and Development	Hematopoiesis of mononuclear leukocytes	1.02E-71	7.192	65
Inflammatory Response	Inflammatory response	3.01E-49	4.939	54
